# Assembly, annotation, and comparative analysis of the first complete mitochondrial genome of *Pennisetum sinese*

**DOI:** 10.3389/fpls.2026.1865296

**Published:** 2026-07-22

**Authors:** Xiaobing Hu, Dan Zhu, Xin Ning, Xiaoyong Shi, Jinwei Zhang, Zhang Zhang, Ran Chai, Biaosheng Lin, Haiwen Zhang, Jiewei Zhang

**Affiliations:** 1 School of Environmental Engineering, Yellow River Conservancy Technical University, Kaifeng, China; 2 State Key Laboratory of Crop Stress Adaptation and Improvement, School of Life Sciences, Henan University, Kaifeng, China; 3 Beijing Key Laboratory of Agricultural Genetic Resources and Biotechnology, Beijing Key Laboratory of Crop Molecular Design and Intelligent Breeding, Beijing Academy of Agriculture and Forestry Sciences, Beijing, China; 4 College of Life Sciences, Longyan University, Longyan, Fujian, China

**Keywords:** chloroplast-derived fragments, mitochondrial genome, *Pennisetum sinese*, phylogenetic analysis, repeat sequences, RNA editing

## Abstract

*Pennisetum sinese*, a perennial grass central to “Juncao Technology,” holds considerable promise for non-grain biomass production and ecological restoration. Despite its agronomic value, the cytoplasmic genetic architecture of this species, particularly its mitochondrial genome, remains uncharacterized. Here, we present the first complete mitochondrial genome of the *P. sinese* assembled *via* hybrid long- and short-read sequencing. The genome adopts a multi-branched conformation spanning 405,186 bp with a GC content of 43.98%, and encodes 32 unique protein-coding genes, 20 tRNA genes, and three rRNA genes. We detected significant codon usage bias, abundant tandem repeats, and dispersed repeats. In addition, 24 chloroplast-derived homologous fragments totaling 13,053 bp were identified. Phylogenetic analysis confirms the placement of the *P. sinese* within the *Poaceae* clade, whereas synteny analysis reveals extensive structural rearrangements in its mitochondrial genome compared with closely related species. Furthermore, we predicted 454 C−to−U RNA editing sites. These findings establish a foundational genetic resource for *P. sinese* cytoplasmic inheritance and laying a foundation for future investigations into the molecular mechanisms underlying its high biomass yield and stress tolerance, informing future germplasm innovation.

## 1 Introduction

*Pennisetum sinese*, native to South America, is a perennial, erect, and tufted herbaceous plant in the genus *Pennisetum* of the *Poaceae* family (Hu et al., 2018). This variety typically reaches a height of over 5 meters and exhibits notable characteristics such as high biomass yield and strong stress tolerance (including drought, poor soil, and saline-alkali conditions), making it one of the core grass species in “Juncao Technology” (He et al., 2020, 2020). Currently, the *P. sinese* has been widely applied in non-grain biomass production, ecological management, and circular agriculture. It is rich in nutrients with a high crude protein content, making it extensively used as forage for livestock (Chen et al., 2025; Liao et al., 2022; Li et al., 2018). Its stems and leaves are abundant in cellulose and hemicellulose, which can replace wood chips and cottonseed hulls in cultivating edible and medicinal fungi, effectively alleviating the “fungi-forest conflict” (Chen et al., 2021). With a dry matter yield of 30-50 t/ha, it serves as high-quality raw material for bioenergy, papermaking, and construction (Cheng et al., 2025). Additionally, its well-developed root system plays a significant role in sand fixation, water retention, mine restoration, and soil improvement (Sun et al., 2021; Wan et al., 2023).

Mitochondria are the primary sites of aerobic respiration in the vast majority of eukaryotic cells, involved in key physiological processes such as energy metabolism, cell apoptosis, and stress response (Jin et al., 2022; Wang et al., 2022). As semi-autonomous organelles, mitochondria possess their own independent genetic material and genetic system (Wu and Tembrock, 2026). The mitochondrial genome is characterized by complex structure, a relatively fast evolutionary rate, and conserved gene content (Cheng et al., 2017; Kozik et al., 2019). Genes encoded by the mitochondrial genome, such as the *atp*, *cox*, and *nad* gene families, are not only crucial to energy metabolism but also serve as an important molecular basis for analyzing species phylogeny, cytoplasmic inheritance patterns, and plant stress resistance mechanisms (Lee et al., 2021; Jin and Daniell, 2015). As such, mitochondrial genomics has become a key target in plant molecular breeding and genetic resource exploration (Wang L, et al., 2024).

Although extensive research has been conducted on the cultivation physiology and ecological applications of the *P. sinese*, its cytoplasmic genetic background, particularly the structure and function of the mitochondrial genome, remains insufficiently systematically characterized (Zheng et al., 2023). Given that the *P. sinese* is a hybrid-bred species, its mitochondria may retain sequences derived from both parental lineages or undergo recombination events. Resolving the structure of its mitochondrial genome is of significant importance for clarifying its cytoplasmic genetic composition (Li X, et al., 2025; Wang J, et al., 2024). To this end, this study assembled and annotated the mitochondrial genome of the *P. sinese*, systematically analyzing its genomic structure, codon usage bias, repetitive sequences, RNA editing sites, sequence migration events, and more. Through comparative genomic analysis with other Poales species, a phylogenetic tree was constructed to elucidate its evolutionary position. This study aims to enrich the genetic information resources of the *P. sinese* and provide a theoretical foundation for uncovering the molecular mechanisms underlying its high biomass and strong stress tolerance, as well as providing genomic clues for future germplasm innovation.

## 2 Materials and methods

### 2.1 Plant materials, DNA extraction and sequence

Plant materials of the *P. sinese*, a perennial grass within the *Poaceae* family, were collected from the greenhouse of the Yellow River conservancy Technical University, located in Kaifeng, City, Henan Province, China. The species was formally identified by Dr. Biaosheng Lin. The mature leaves were collected, snap frozen in liquid nitrogen, and stored in a -80°C refrigerator. Total genomic DNA was extracted using the FastPure^®^ Plant DNA Isolation Mini Kit (DC104, Vanzyme Biotech, Nanjing, China) according to the manufacturer’s protocol. The DNA was subsequently sequenced using both Nanopore long-read and next-generation sequencing (NGS) technologies, following the corresponding sequencing library preparation protocols.

### 2.2 Mitochondrial genome assembly

First, assemble the mitochondrial genome based on long-read data. The long-read sequencing data were directly assembled using Flye software (v2.9.2-b1786) (Kolmogorov et al., 2019) with default parameters, yielding a graphical assembly result in GFA format. For all assembled contigs in FASTA format, a BLAST database was constructed using makeblastdb. Subsequently, the BLASTn program was employed to identify contig fragments containing mitochondrial genomes, using the mitochondrial genomes of closely related species (Cenchrus fungigraminus, PQ720776.1; Setaria italica, OR734218.1-OR734219.1) as query sequences, with parameters set as “-evalue 1e-5 -outfmt 6 -max_hsps 10 -word_size 7 -task blastn-short”. The GFA file was visualized using Bandage software (v0.8.1) (Wick et al., 2015), and mitochondrial contigs were screened based on the BLASTn results, resulting in a draft mitochondrial genome.

Subsequently, error correction of the mitochondrial genome was performed using second-generation data with Fmlrc2 (v0.1.7) (Mak et al., 2023), Ropebwt2 (vr187) (Li, 2014), and Msbwt (v0.2.9) (Holt and McMillan, 2014). The specific procedure was as follows: First, index files, including msbwt.npy, were generated using Msbwt and Ropebwt2 with the command line “gunzip -c $Illumina_1r1 $Illumina_1r2 | awk ‘NR % 4 = 2’ | sort | tr NT TN | ropebwt2 -LR | tr NT TN | msbwt convert/output/”, where $Illumina_1r1and $Illumina_1r2 represent the paired-end reads, and msbwt.npy was generated in the/output/directory. Thereafter, error correction was performed using Fmlrc2 with the command line “fmlrc -t 20 msbwt.npy assembled_mtgenome.fasta corrected_mtgenome.fasta”. Finally, the corrected mitochondrial genome was obtained.

### 2.3 Gene annotation

The mitochondrial genome was annotated using the angiosperm mitochondrial genome annotation tool PMGA (http://www.1kmpg.cn/pmga/) (Li J, et al., 2025), with the reference database set to 319 mitochondrial genomes. PMGA demonstrated good performance in annotating splice sites and trans-splicing genes. tRNAs in the mitochondrial genome were annotated using tRNAscan-SE software (v2.0.11) (Lowe and Eddy, 1997). rRNAs in the mitochondrial genome were annotated using BLASTN software (v2.16.0) (Chen et al., 2015). Annotation errors in the mitochondrial genome were manually corrected using Apollo software (v1.11.8) (Lewis et al., 2002). The mitochondrial genome map was visualized using OGDRAW software (Greiner et al., 2019).

### 2.4 Codon usage bias analysis

The protein-coding sequences of the mitochondrial genome were extracted using Phylosuite software (v1.1.16) (Zhang et al., 2020). Codon usage bias analysis was performed on the protein-coding genes (PCGs) of the mitochondrial genome with MEGA software (v7.0) (Kumar et al., 2016). The Relative Synonymous Codon Usage (RSCU) values were calculated accordingly.

### 2.5 Repetitive sequence analysis

Tandem repeats, microsatellites, and interspersed repeats were identified using MISA (v2.1) (Beier et al., 2017), Tandem Repeats Finder (TRF, v4.09) (Benson, 1999), and Rousfinder (Wynn and Christensen, 2019), respectively. The results were visualized using Excel (2021) and the Circos package (v0.69.9) (Zhang et al., 2013).

### 2.6 Sequence migration analysis

Based on short-read sequencing data, the chloroplast genome was assembled using GetOrganelle (v1.7.7.0) (Jin et al., 2020), annotated with CPGAVAS2 (Shi et al., 2019), and subsequently corrected for annotation accuracy with CPGView (Liu et al., 2013). Homologous fragments were analyzed using BLASTN (v2.16.0) (Chen et al., 2015), and the results were visualized with the Circos package (v0.69.9) (Zhang et al., 2013).

### 2.7 Phylogenetic analysis

Based on phylogenetic relationships, mitochondrial genomes of closely related species were selected and downloaded. Shared genes were extracted using PhyloSuite software (v1.1.16) (Zhang et al., 2020), followed by multiple sequence alignment performed with MAFFT software (v7.525) (Katoh and Standley, 2013). A maximum likelihood phylogenetic tree was then constructed using IQ-TREE software (v2.3.6) (Minh et al., 2020) with the parameters “--alrt 1000 -B 1000”. The results of the phylogenetic analysis were visualized using Interactive Tree of Life (iTOL) v7 (Letunic and Bork, 2024).

### 2.8 Synteny analysis

Conserved homologous sequences were first identified using BLASTn (-evalue 1e-5 -outfmt 6 -perc_identity 80). Syntenic blocks were then detected and extracted from these BLASTn hits using MCscanX (Wang et al., 2012), with only blocks longer than 500 bp retained for downstream analysis.

### 2.9 RNA editing events analysis

All protein-coding gene (PCG) sequences encoded in the mitochondrial genome of the *P. sinese* were used as input. C-to-U RNA editing sites in mitochondrial PCGs were predicted using Deepred-mt (Edera et al., 2021), a tool based on a convolutional neural network (CNN) model that demonstrates higher accuracy compared to previous prediction tools. All results with a probability value greater than 0.9 were retained.

## 3 Results

### 3.1 Assembly and annotation of the mitochondrial genome

**Figure 1:**
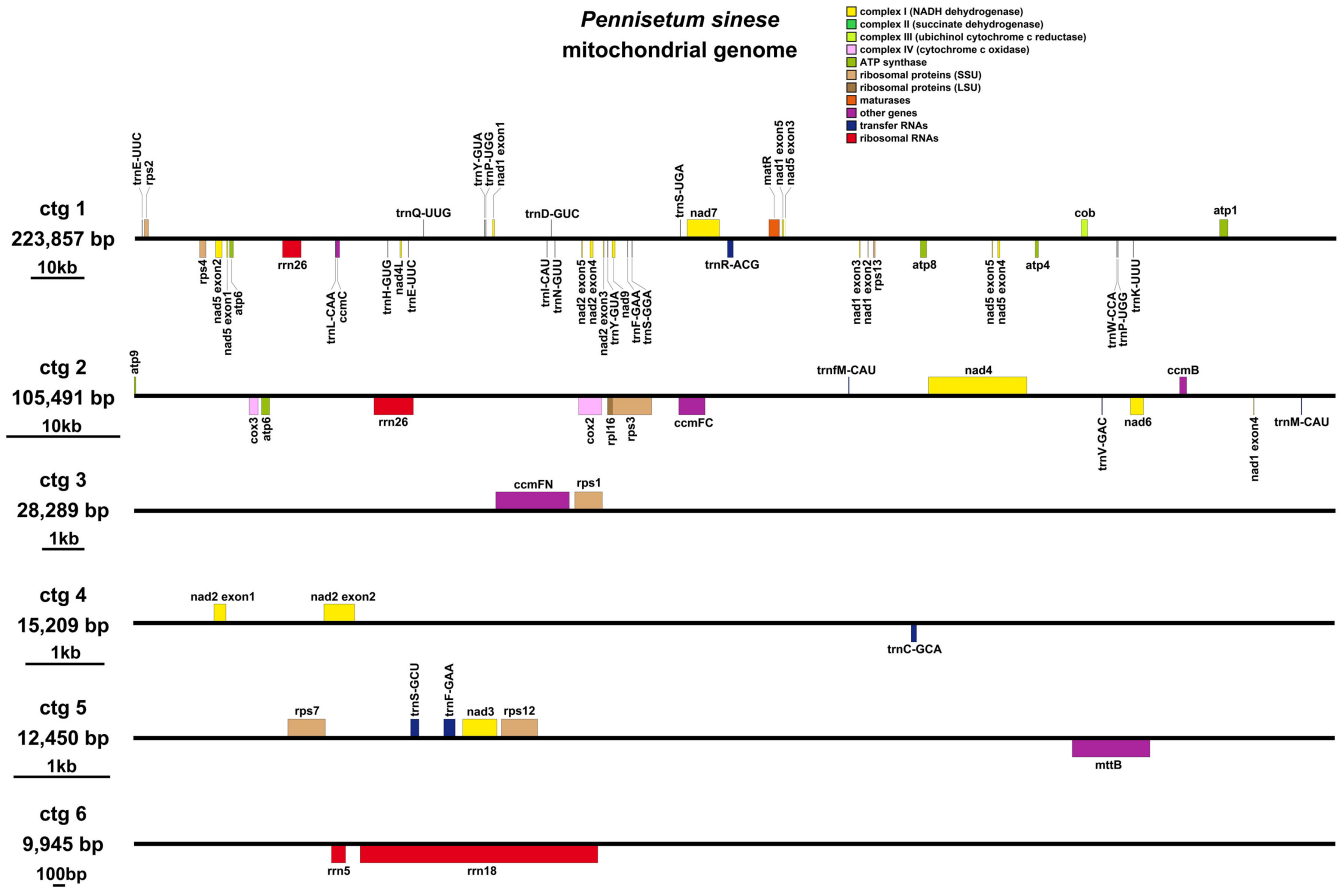
Mitochondrial genome map of the *P. sinese.* Linearized map of the circular mitochondrial genome of the *C. fungigraminus.* A horizontal black line indicates the linearized axis of the genome. The colored rectangles, lines, and symbols above the axis represent the positions, lengths, and transcriptional directions of annotated features. Colors correspond to functional categories shown in the legend on the right.

Plant mitochondrial genomes can exist in various conformations, including circular, multi−circular, linear, and multi−branched structures. In most cases, however, plant mitochondrial genomes are maintained as a single circular molecule, which can be fragmented into multiple smaller circles through recombination events mediated by repetitive sequences. For linear plant mitochondrial genome structures, hybrid assembly of the mitochondrial genome can restore the closed multi−branched configuration of the linear molecule, thereby confirming its completeness. The primary structure of the *P. sinese* mitochondrial genome exhibits a multi−branching configuration, with a total length of 405, 186 bp and a GC content of 43.98 % (**Figure 1**).

The draft mitochondrial genome of the *P. sinese*, assembled from long-read data, was visualized using Bandage software (v0.8.1). The final assembly graph consists of six contigs connected to form a branched structure (**Supplementary Figure 1A**). To validate the linkages between these contigs, reference sequences were generated by extracting 2, 000 bp from each overlapping region between adjacent nodes. Long reads were then mapped to each reference sequence to count the number of reads supporting each linkage. As summarized in **Supplementary Table 1**, all linkages were supported by long-read evidence. Based on the validated connections, the mitochondrial genome structure was linearized in the order ctg3-ctg2-ctg6-ctg1-ctg5-ctg6_copy-ctg4, resulting in a single linear sequence representing the complete mitochondrial genome of this species (**Supplementary Figure S1B**). Information corresponding to each final sequence and its associated node in **Supplementary Figure S1A** is summarized in **Supplementary Table S2**. Furthermore, to experimentally verify the connectivity and completeness of the assembly, specific primers flanking all eight contig junction regions were designed and PCR amplification was performed, followed by Sanger sequencing (**Supplementary Figure 3**). All eight junctions successfully amplified fragments of the expected sizes, and the Sanger sequencing results confirmed the assembled contig junction sequences. This experimental evidence unequivocally demonstrates that the connections between all contigs are genuine, supporting the completeness and accuracy of the reported mitogenome.

Annotation of the *P. sinese* mitochondrial genome identified a total of 32 unique protein−coding genes (including 2 multi−copy genes), comprising 24 unique mitochondrial core genes and 8 non−core genes, 20 tRNA genes (including 4 multi−copy genes), and 3 rRNA genes (all 3 of which are multi−copy). Core genes are defined as those highly conserved across angiosperms and essential for respiratory chain function and mitochondrial gene expression. In contrast, non-core genes are variable among species and mainly consist of ribosomal protein genes often acquired via intracellular or horizontal gene transfer (Kozik et al., 2019). The core genes include 5 ATP synthase genes (*atp1*, *atp4*, *atp6*, *atp8*, and *atp9*); 9 NADH dehydrogenase genes (*nad1*, *nad2*, *nad3*, *nad4*, *nad4L*, *nad5*, *nad6*, *nad7*, and *nad9*); 4 cytochrome c biogenesis genes (*ccmB*, *ccmC*, *ccmFC*, and *ccmFN*); 3 cytochrome c oxidase genes (*cox1*, *cox2*, and *cox3*); 1 membrane transport protein gene (*mttB*); 1 maturase gene (*matR*); and 1 cytochrome b gene (*cob*). The non−core genes include 1 large ribosomal subunit gene (*rpl16*) and 7 small ribosomal subunit genes (*rps1*, *rps2*, *rps3*, *rps4*, *rps7*, *rps12*, and *rps13*) (**Table 1**).

**Table 1:** Protein-coding genes in the mitochondrial genome of *Pennisetum sinese*.

Group of genes	Name of genes
ATP synthase	*atp*1,*atp*4,*atp*6(×2),*atp*8,*atp*9
NADH dehydrogenase	*nad*1,*nad*2,*nad*3,*nad*4,*nad*4L,*nad*5,*nad*6,*nad*7,*nad*9
Cytochrome *b*	*cob*
Cytochrome *c* biogenesis	*ccm*B,*ccm*C,*ccm*FC,*ccm*FN
Cytochrome *c* oxidase	*cox*1(×2),*cox*2,*cox*3
Maturases	*mat*R
Protein transport subunit	*mtt*B
Ribosomal protein large subunit	*rpl*16
Ribosomal protein small subunit	*rps*1,*rps*2,*rps*3,*rps*4,*rps*7,*rps*12,*rps*13
Ribosomal RNA	*rrn*5(×2),*rrn*18(×2),*rrn*26(×2)
Transfer RNA	*trn*C-GCA,*trn*D-GUC,*trn*E-UUC(×2),*trn*F-GAA(×2),*trnf*M-CAU,*trn*H-GUG,*trn*I-CAU,*trn*K-UUU,*trn*L-CAA,*trn*M-CAU,*trn*N-GUU,*trn*P-UGG(×2),*trn*Q-UUG,*trn*R-ACG,*trn*S-GCU,*trn*S-GGA,*trn*S-UGA,*trn*V-GAC,*trn*W-CCA,*trn*Y-GUA(×2)

### 3.2 Analysis of codon usage bias in the mitochondrial genome

**Figure 2:**
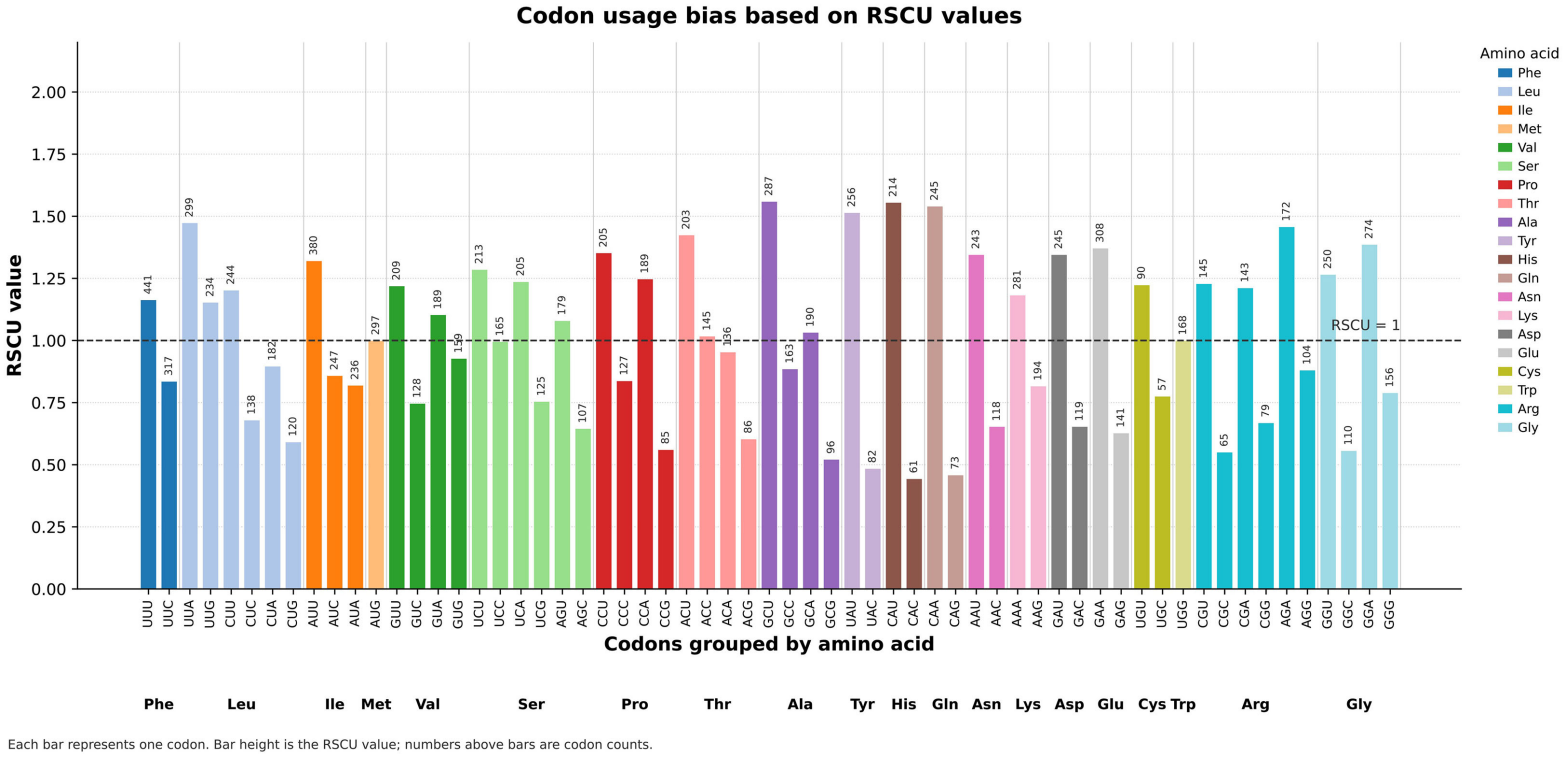
Analysis of codon usage bias in the *P. sinese* mitochondrial genome. The X-axis represents codon families, while RSCU values indicate the frequency of a specific codon relative to the expected frequency under uniform synonymous codon usage.

Eukaryotic genomes encode 64 codons, which specify 20 different amino acids and 3 stop codons. With the exception of methionine and tryptophan, all amino acids are encoded by multiple codons. Significant variations in codon usage frequency are observed across different species and organisms. This bias is considered to result from long-term evolutionary selection, through which cells have gradually established a relatively balanced state. Codon usage bias analysis was performed on the PCGs of the *P. sinese* mitochondrial genome. The usage patterns of codons for each amino acid are presented in **Supplementary Table 3**. Codons with a RSCU value greater than 1 are considered preferentially used by the corresponding amino acid. As shown in **Figure 2**, the codon usage bias of mitochondrial PCGs is quantified by RSCU values, with codons grouped by their corresponding amino acids. In the figure, each bar represents one codon; the height of the bar corresponds to its RSCU value, and the number above each bar indicates the actual codon count observed in the dataset. Apart from the start codon AUG and tryptophan (UGG), both of which have an RSCU of exactly 1, a clear bias is evident across all amino acids. For example, alanine (Ala) displays a marked preference for the codon GCU, which reaches the highest RSCU (1.56) among all mitochondrial PCGs, and its elevated codon count is also visible above the corresponding bar.

### 3.3 Analysis of repetitive sequences in the mitochondrial genome

**Figure 3:**
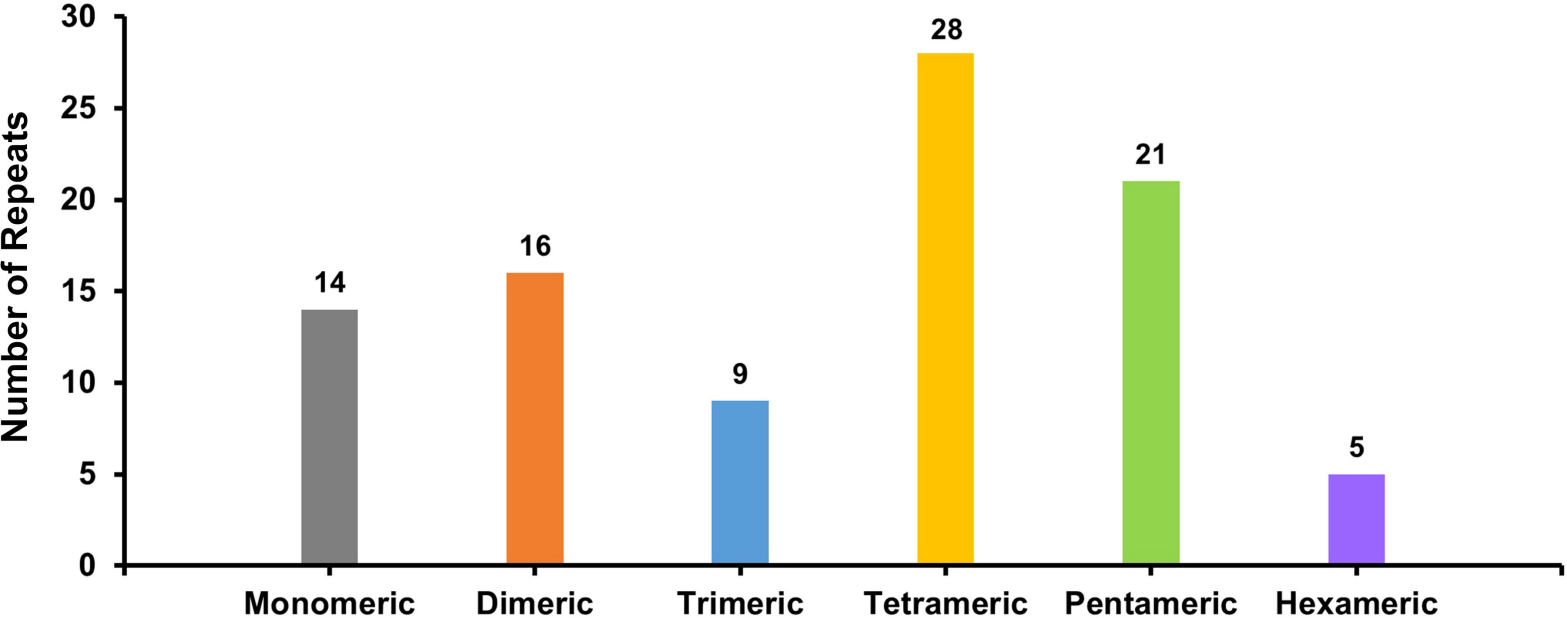
Schematic representation of SSRs distribution in the *P. sinese*. The X-axis denotes mitochondrial molecules, while the Y-axis indicates the frequency of repetitive fragments. Monomeric SSRs are denoted by gray, dimeric SSRs by orange, trimeric SSRs by blue, tetrameric SSRs by yellow, pentameric SSRs by green, and hexameric SSRs by purple.

DNA sequences in biological cells contain numerous repetitive sequences, which can be broadly classified into two main categories: tandem repeats and dispersed repeats. Tandem repeats, also referred to as satellite DNA, consist of core repeat units of approximately 2-200 base pairs repeated consecutively multiple times. They are widely distributed in eukaryotic and prokaryotic genomes. In the mitochondrial genome of the *P. sinese*, a total of 38 tandem repeat sequences were identified with similarity greater than 78% and lengths ranging from 5 to 49 bp (**Supplementary Table 4**). Among tandem repeats, simple sequence repeats (SSRs) represent a specific type of short tandem repeat, typically with repeat unit lengths not exceeding 6 bp. Due to their characteristics such as codominant inheritance, SSRs are commonly utilized in molecular marker development. In the mitochondrial genome of the *P. sinese*, 93 SSRs were identified, with tetrameric SSRs constituting 30.11% (28) of the total. Among monomeric SSRs, thymine (T) repeats accounted for 50.00% (7 out of 14) (**Figure 3**) (**Supplementary Table 5**).

Dispersed repeats in the mitochondrial genome of the *P. sinese* were also examined. A total of 2, 773 dispersed repeat sequences with lengths ≥ 50 bp were detected, including 1, 047 palindromic repeats and 1, 726 forward repeats. The longest dispersed repeat unit, designated as R1, measured 17, 550 bp in length (**Supplementary Table 6**).

### 3.4 Analysis of sequence migration

**Figure 4:**
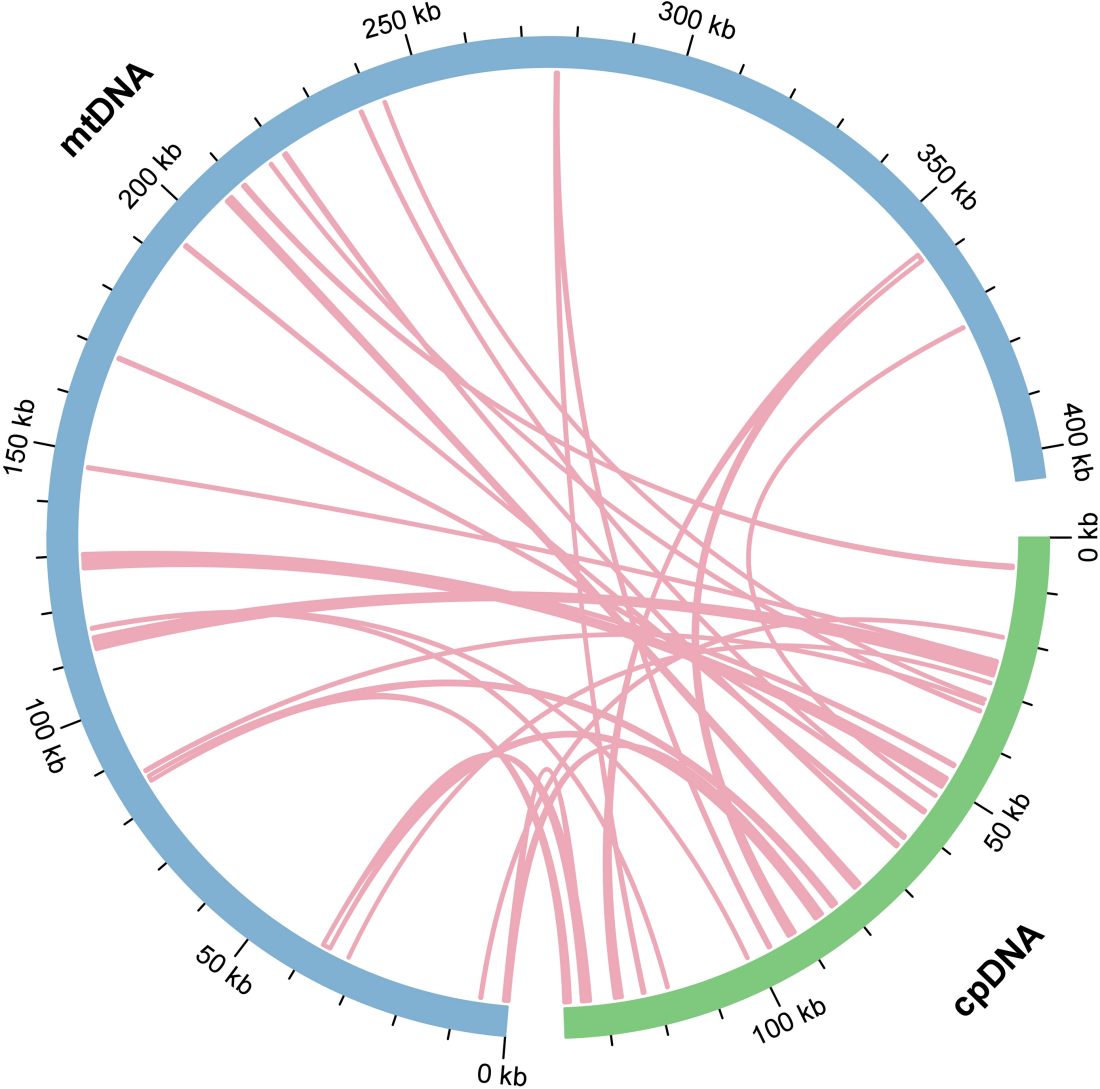
Schematic diagram of gene transfer between chloroplast and mitochondrial genomes in the *P. sinese*. The blue arc represents the mitochondrial genome, the green arc represents the chloroplast genome, and the pink connecting lines between the arcs correspond to homologous genomic fragments.

During mitochondrial evolution, fragments of chloroplast DNA can be transferred into the mitochondrial genome. The length and sequence similarity of these transferred fragments vary among different species. Based on sequence similarity analysis, a total of 24 homologous fragments were identified between the mitochondrial and chloroplast genomes of the *P. sinese*, with a combined length of 13, 053 bp, accounting for 3.22 % of the mitochondrial genome. The longest of these fragments, designated MTPT1, spans 2, 287 bp. Annotation of these homologous sequences revealed 13 complete genes within the 24 fragments, including 3 protein-coding genes (*ndhJ*, *psbE*, *ycf15*) and 10 tRNA genes (*trnC-GCA*, *trnF-GAA*, *trnH-GUG*, *trnM-CAU*, *trnN-GUU*, *trnP-GGG*, *trnP-UGG*, *trnS-GGA*, *trnT-GGU*, *trnW-CCA*) (**Figure 4**) (**Supplementary Table 7**).

### 3.5 Phylogenetic and synteny analysis

A phylogenetic tree was constructed based on the DNA sequences of 25 conserved mitochondrial PCGs from 42 species within the order Poales (**Supplementary Table 9**). The shared PCGs included *atp1*, *atp4*, *atp6*, *atp8*, *atp9*, *ccmB*, *ccmC*, *ccmFC*, *ccmFN*, *cox1*, *cox2*, *matR*, *mttB*, *nad1*, *nad2*, *nad3*, *nad5*, *nad6*, *nad7*, *nad9*, *rps1*, *rps2*, *rps4*, *rps7*, and *rps13*.

Based on mitochondrial conserved protein-coding genes from 42 species (covering *Panicoideae*, *Chloridoideae*, *Oryzoideae*, *Pooideae*, *Bambusoideae* within Poaceae, and *Cyperoideae* within Cyperaceae), the phylogenetic topology is consistent with the latest Angiosperm Phylogeny Group (APG) classification, firmly placing *P. sinese* within the subfamily Panicoideae of the family Poaceae. Beyond this placement, the analysis further reveals a specific and strongly supported close relationship between *P. sinese* and its congeners *Cenchrus americanus* and *C. fungigraminus* (bootstrap support = 100%). This finding aligns with recent phylogenetic studies advocating the taxonomic consolidation of *Pennisetum* and *Cenchrus* into a single genus. Collectively, our results confirm that *P. sinese* is deeply nested within the *Cenchrus* clade, rather than representing a distinct lineage outside it.

**Figure 5:**
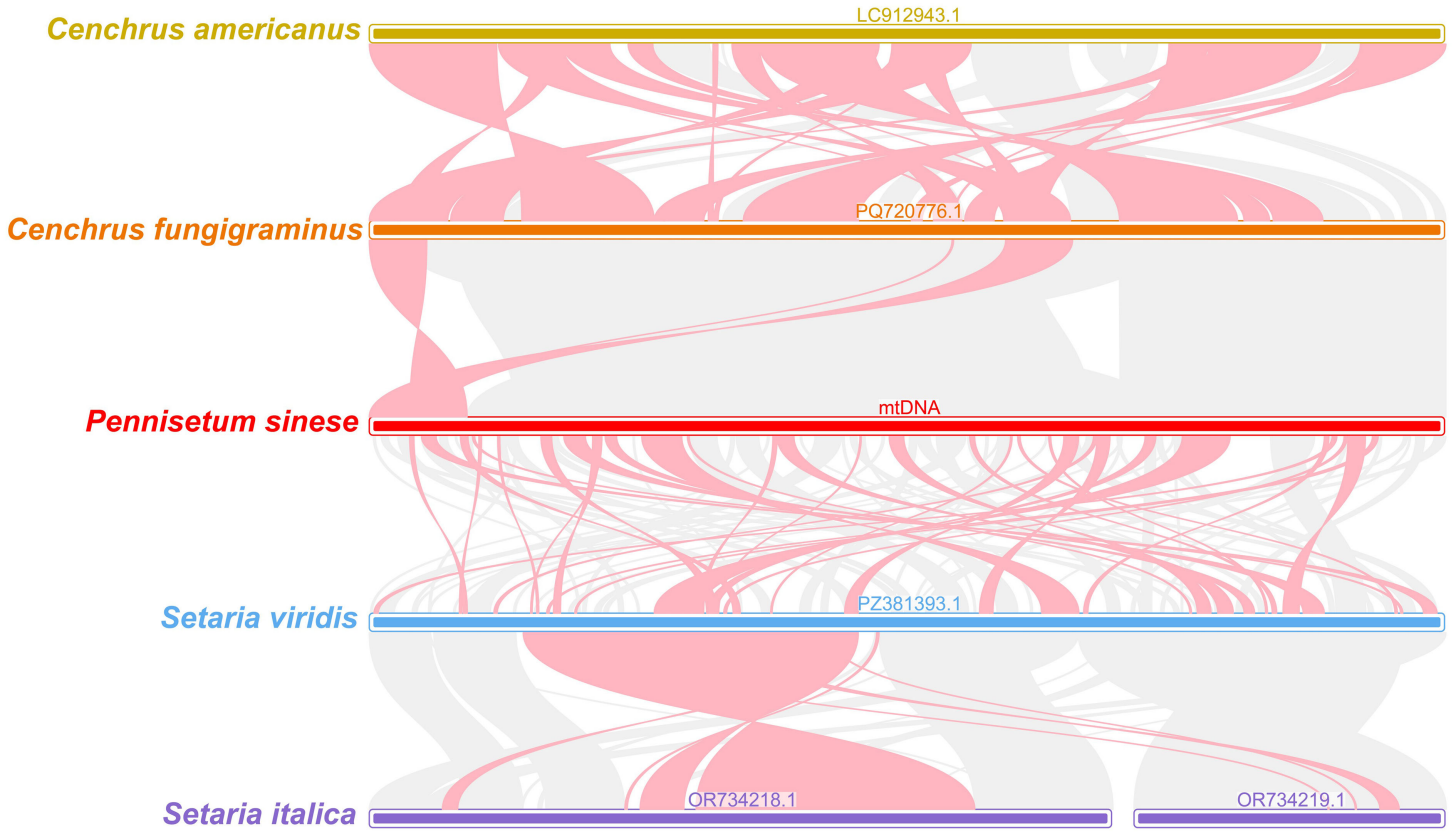
Synteny analysis of the *P. sinese* mitochondrial genome. The red arc regions represent areas of inversion, while the gray regions indicate regions with good homology. To better present the results, syntenic blocks shorter than 0.5 kb were not retained in the output.

Synteny analysis, which refers to the arrangement of homologous genes or sequences, is commonly used to evaluate genome assembly quality, gene retention and loss, and evolutionary relationships. As shown in **Figure 5**, numerous homologous syntenic blocks were detected between the *P. sinese* and its closely related species. While the presence of these blocks confirms sequence-level homology, the order of these blocks is markedly different in the *P. sinese* compared to its relatives, indicating extensive genome rearrangement.

**Figure 6:**
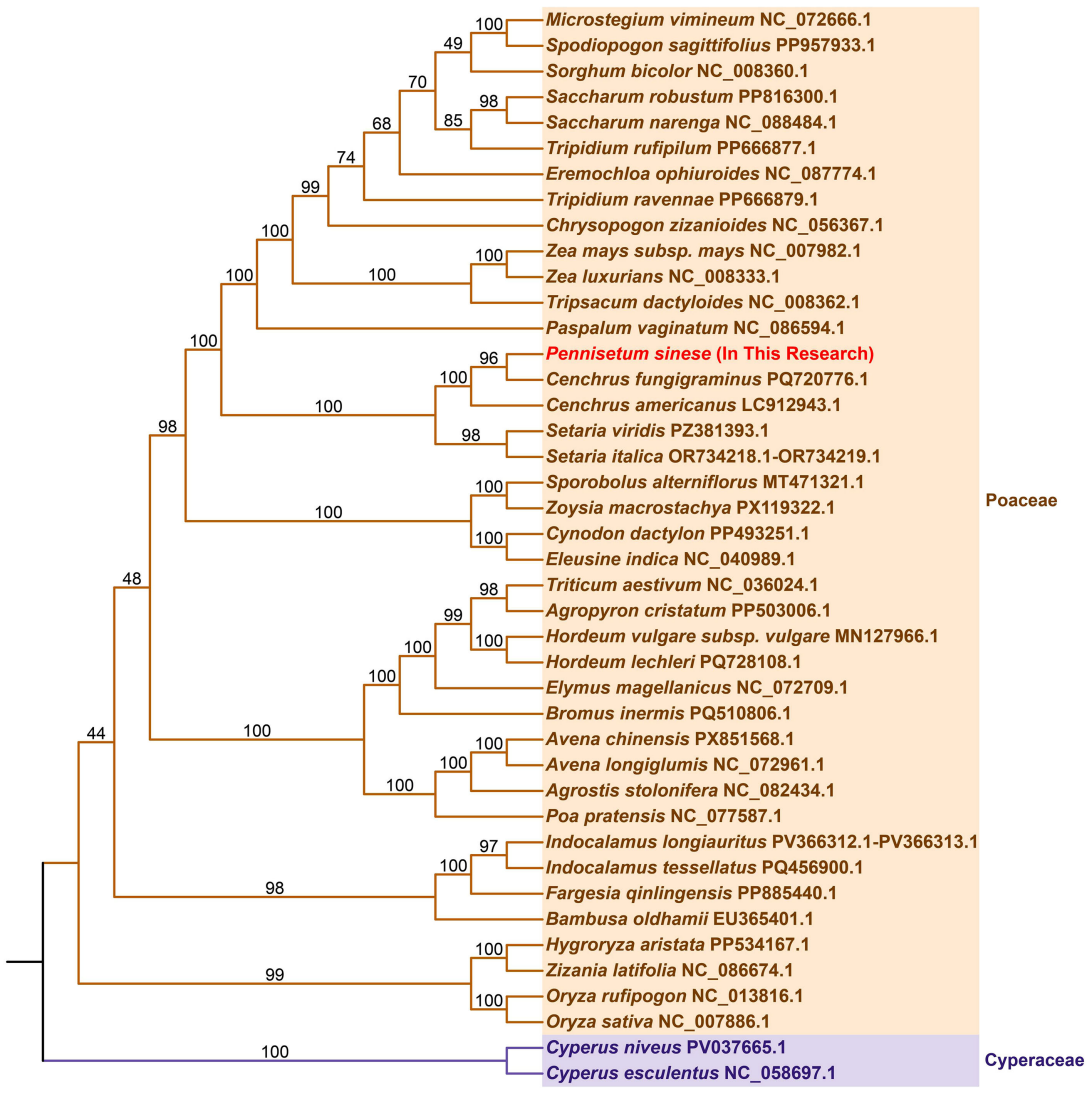
Phylogenetic analysis of the *P. sinese* mitochondrial genome. Bootstrap support values are shown on each node, with colors representing the respective plant families.

Given the close phylogenetic affinity shown in **Figure 6**, where *P. sinese* forms a closely related clade with *C. fungigraminus*, the extensive structural rearrangements observed between these two taxa are particularly striking. This marked contrast suggests a rapid evolution of the mitochondrial genome within this specific lineage of the *Cenchrus*, despite their relatively recent divergence.

### 3.6 Analysis of RNA editing events

**Figure 7:**
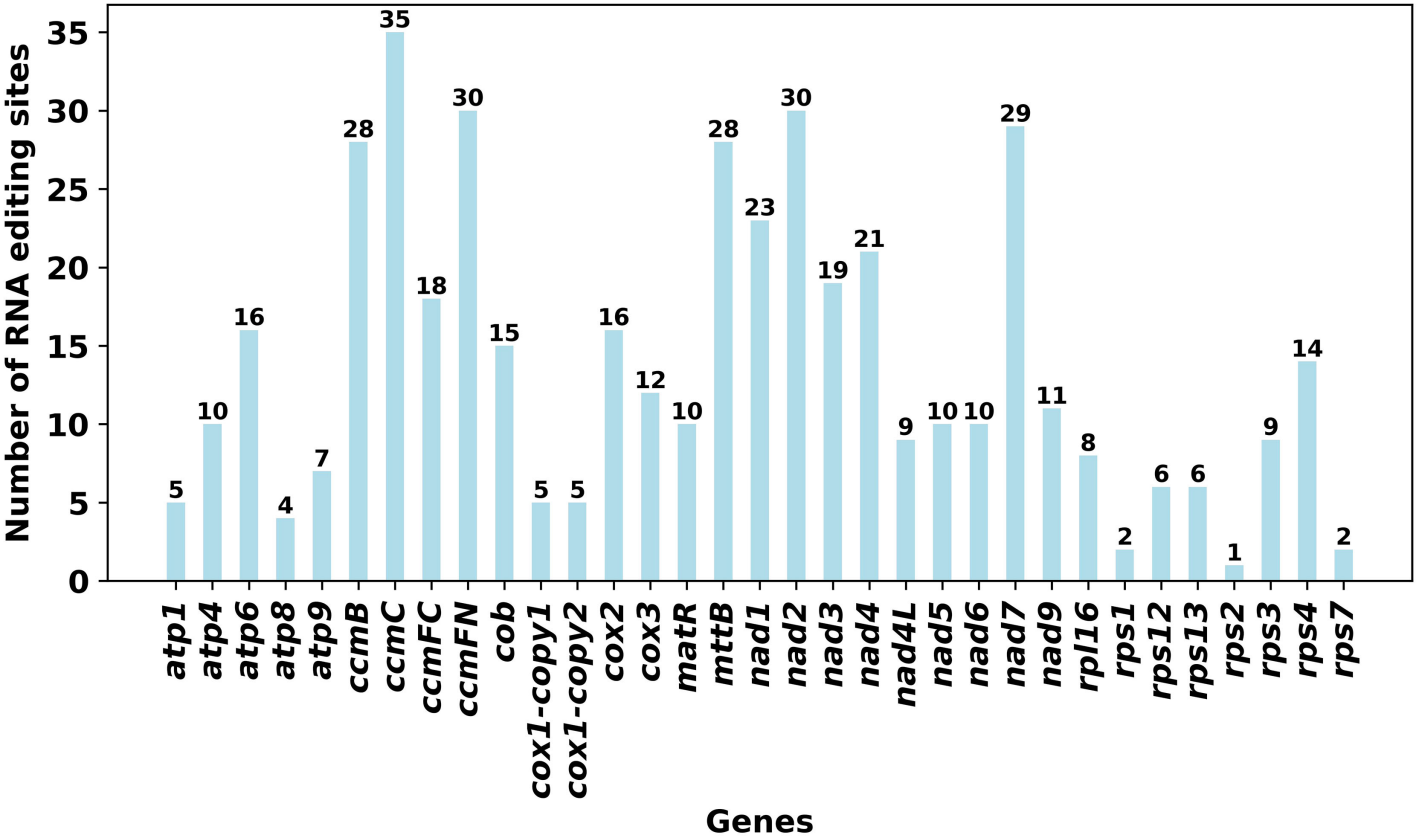
Schematic representation of predicted RNA editing events in the *P. sinese* mitochondrial genome.

Potential RNA editing sites in the PCGs of the *P. sinese* mitochondrial genome were identified using a cutoff value of 0.9. Under this criterion, a total of 454 potential RNA editing sites, all representing C-to-U conversions, were detected. Among these, the majority occur at the second codon position (e.g., UCA→UUA, CCA→CUA, CCG→CUG), while a smaller number occur at the first codon position (e.g., CUC→UUC, CCC→UCC). Editing at the third codon position was rarely observed, as such changes would typically be synonymous and are not listed in the **Supplementary Table 8**. Among the mitochondrial genes, *ccmC* contained the highest number of RNA editing sites (35), followed by *ccmFN* and *nad2*, each with 30 identified editing sites (**Figure 7**). Furthermore, in **Supplementary Table 8**, we identified a highly conserved editing event in the *ccmFC* gene, an efficient C-to-U editing (probability > 0.99) occurring at the 1312th nucleotide.

## 4 Discussion

This study presents the first bioinformatics analysis of the complete mitochondrial genome of the *P. sinese*. The results not only reveal the fundamental characteristics and evolutionary dynamics of its mitochondrial genome but also provide new insights into the evolution of organellar genomes within the genus *Cenchrus* the *Poaceae* family as a whole.

### 4.1 Complexity of genome structure

The mitochondrial genome of the *P. sinese* exhibits a typical multi-branched structure, a common feature of plant mitochondrial genomes (Wang et al., 2025; Xiao et al., 2025). This structural complexity arises from homologous recombination mediated by repetitive sequences, which can lead to the fragmentation of the primary circular molecule into multiple subgenomic circles. The hybrid assembly strategy employed in this study successfully reconstructed its linearized “master path” structure, confirming the completeness and accuracy of the assembly. This inherent structural complexity may serve as a foundation for both genetic stability and evolutionary plasticity.

### 4.2 Genomic imprints of hybrid origin

As a cultivar developed through distant hybridization, elucidating its mitochondrial genome is crucial for understanding its cytoplasmic genetic composition. Although the assembly in this study yielded a single consistent sequence, subsequent re-sequencing of the parental genomes or more in-depth local assembly analyses may enable the direct detection of potential parental-derived fragments or recombination traces at the sequence level. Such findings would provide a valuable case study for understanding the evolutionary fate of cytoplasmic genomes in hybrid species.

### 4.3 Potential links to high biomass production

The mitochondrial genome of the *P. sinese* encodes a complete set of genes involved in energy metabolism (e.g., the *atp*, *nad*, and *cox* families). Further investigation into the expression patterns of these genes, particularly their coordinated expression with nuclear-encoded subunits, may unravel the regulatory networks underlying efficient energy metabolism. This represents a key cellular basis for its rapid growth and high biomass accumulation.

### 4.4 Molecular clues to stress tolerance

C-to-U RNA editing is a widespread phenomenon in higher plant mitochondria, representing a crucial post-transcriptional modification (Liao et al., 2026). This process often alters amino acid sequences-converting hydrophilic residues to hydrophobic ones to promote proper protein folding-and can generate start or stop codons, thereby enhancing protein conservation across species. In the context of the *P. sinese*, we identified 454 potential C-to-U editing sites, all representing C-to-U conversions.

Mitochondria are critical organelles for sensing and responding to environmental stresses such as drought and salinity. The large number of RNA editing sites identified in this study, particularly in genes like *ccmC*, *ccmFN*, and *nad2*, may fine-tune mitochondrial function at the post-transcriptional level by altering protein amino acid sequences, thereby influencing protein structure and activity to facilitate adaptation to stress conditions. A particularly noteworthy finding is a highly conserved C-to-U RNA editing event (editing probability > 0.99) identified in the *ccmFC* gene at nucleotide position 1312, which converts the CAA (glutamine) codon into a UAA stop codon. This type of editing is widespread in land plants and constitutes an important post-transcriptional regulatory mechanism. For example, in rice, mutations in *ccmFC* editing factors result in severe dwarfism (Hou et al., 2022), whereas in *Sophora moorcroftiana*, analogous editing has been directly linked to drought tolerance (Xu et al., 2025). These observations collectively suggest that this editing event may play a potential role in maintaining normal plant growth and stress adaptation.

The *P. sinese* exhibits rapid growth, high biomass accumulation, and enhanced stress resilience-including tolerance to drought, nutrient-poor soils, and saline-alkaline conditions-all of which rely heavily on a sustained energy supply. The highly efficient RNA editing in the *ccmFC* gene is hypothesized to contribute to these traits by ensuring proper cytochrome csynthesis, which helps maintain the structural integrity and operational efficiency of the mitochondrial electron transport chain (ETC). This optimization maximizes ATP production, providing the energetic foundation for vigorous growth. Moreover, a robust ETC supported by this editing can sustain ATP supply under stress, thereby supporting energy-demanding adaptive responses such as osmotic adjustment and reactive oxygen species (ROS) scavenging, while also reducing electron leakage and minimizing oxidative damage at the source-key mechanisms underlying its drought and salt tolerance. In summary, we propose that the efficient RNA editing in the *ccmFC* gene represents a critical molecular basis for the superior agronomic performance of the *P. sinese*, including its high biomass yield and multi-stress resistance. By securing the efficiency of core energy-metabolism pathways, this editing event underpins both rapid growth and stress adaptation. Our findings provide new clues to the potential “energy-metabolism optimization” mechanism in this species.

It is important to note that this study relies on *in silico* predictions for RNA editing sites. While Deepred-mt provides high accuracy, experimental validation via RT-PCR or Sanger sequencing of cDNA is necessary to confirm the editing efficiency and functional consequences of these sites, particularly for the *ccmFC* locus.

### 4.5 Evolution and phylogenetic position

Our mitochondrial phylogenomics results provide critical insights into the taxonomic delimitation of the *Pennisetum* and *Cenchrus*. While morphological traits have historically separated these genera, recent molecular evidence, including the chromosome-level genome of the *C. fungigraminus* (Zheng et al., 2023), supports their unification. The fact that the mitochondrial genome of the *P. sinese* clusters with *C. fungigraminus* to the exclusion of other *Pennisetum* species (such as *P. glaucum*) suggests that *P. sinese*is deeply nested within the *Cenchrus* clade. This close relationship explains the high sequence similarity observed in our comparative analyses and underscores the need for a unified taxonomic treatment of these high-biomass grasses.

Based on a phylogenetic tree constructed from mitochondrial conserved protein-coding genes of 42 species (covering *Panicoideae, Chloridoideae, Oryzoideae, Pooideae, Bambusoideae, and Cyperaceae*), *P. sinese* is unequivocally placed within the Panicoideae clade of *Poaceae*, clustering closely with its congeners such as *C. americanus* and *C. fungigraminus*. This result is highly consistent with previous classifications based on morphological and nuclear gene data, thereby further confirming its taxonomic status from the perspective of the cytoplasmic genome. Meanwhile, synteny analysis reveals extensive genomic rearrangements within this clade and among different subfamilies of *Poaceae*, indicating that although mitochondrial gene content is relatively conserved, its structure exhibits remarkable plasticity in *Poaceae*; such rearrangements may serve as an important driving force for species differentiation and diversification.

## 5 Conclusion

This study successfully accomplished the *de novo* assembly, annotation, and comprehensive analysis of the first complete mitochondrial genome of the *P. sinese*. A high-quality mitochondrial genome sequence was obtained, exhibiting a typical multi-branched structure common in plant mitochondria, with a total length of 405, 186 bp and containing 55 annotated genes (including PCGs, tRNAs, and rRNAs). Genomic analyses revealed its distinctive codon usage pattern, an abundance of repetitive sequences (38 tandem repeats and 2, 773 dispersed repeats), and chloroplast-derived migration events (24 fragments, accounting for 3.22% of the genome). Phylogenetic analysis clarified the taxonomic position of the *P. sinese* within the genus *Cenchrus*, meanwhile synteny analysis indicated that its genome structure has undergone rearrangement. Furthermore, 454 potential C-to-U RNA editing sites were predicted, and it was discovered that the *ccmFC* gene undergoes premature termination due to RNA editing, providing new molecular clues for understanding the formation of its excellent agronomic traits. In summary, this study provides the first complete mitochondrial genome of *P. sinese*. Multi-faceted comparative genomic analyses establish a foundational resource for elucidating the molecular basis of its high biomass and stress tolerance, thereby informing future molecular breeding strategies based on cytoplasmic inheritance.

## Data Availability

The datasets presented in this study can be found in online repositories. The names of the repository/repositories and accession number(s) can be found in the article/**Supplementary Material**.
