# Supplementary material for: Assembly, annotation, and comparative analysis of the first complete mitochondrial genome of Pennisetum sinese
Source: Front Plant Sci. 2026 Jul 22;17:1865296. doi: 10.3389/fpls.2026.1865296 (PMC13438385; doi:10.3389/fpls.2026.1865296)
Supplement: Supplementary file 1 [file DataSheet1.docx]

Figure S1. Schematic representation of the *P. sinese* mitochondrial genome assembly.

Figure S2 Whole-plant morphology of *P. sinese* and *C. fungigraminus.*

Figure S3. Agarose gel electrophoresis validation of the eight contig junctions in the *P. sinese* mitochondrial genome. All eight junctions yielded PCR products of the expected sizes, confirming the connectivity between adjacent contigs. M, DNA marker.
